# Assessing Sensory Processing Dysfunction in Adults and Adolescents with Autism Spectrum Disorder: A Scoping Review

**DOI:** 10.3390/brainsci7080108

**Published:** 2017-08-19

**Authors:** Denise DuBois, Erin Lymer, Barbara E. Gibson, Pushpal Desarkar, Emily Nalder

**Affiliations:** 1 Rehabilitation Sciences Institute, Faculty of Medicine, University of Toronto, Toronto, ON M5G 1V7, Canada; 2 Adult Neurodevelopmental Service, Centre for Addiction and Mental Health, Toronto, ON M6J 1H4, Canada; erin.lymer@camh.ca (E.L.); pushpal.desarkar@camh.ca (P.D.); barbara.gibson@utoronto.ca (E.N.); 3 Department of Occupational Science and Occupational Therapy, Faculty of Medicine, University of Toronto, Toronto, ON M5G 1V7, Canada; emily.nalder@utoronto.ca; 4 Department of Physical Therapy, Faculty of Medicine, University of Toronto, Toronto, ON M5G 1V7, Canada; 5 Bloorview Research Institute, Holland Bloorview Kids Rehabilitation Hospital, Toronto, ON M4G 1R8, Canada; 6 Department of Psychiatry, Faculty of Medicine, University of Toronto, Toronto, ON M5T 1R8, Canada; 7 Temerty Centre for Therapeutic Brain Intervention, Centre for Addiction and Mental Health; Toronto, ON M6J 1H4, Canada; 8 March of Dimes Canada, Toronto, ON M4H 1A4, Canada

**Keywords:** sensory processing, sensory reactivity, autism spectrum disorder, clinical guidelines, interdisciplinary, best practice, evidence-based practice, adult

## Abstract

Sensory reactivity is a diagnostic criterion for Autism Spectrum Disorder (ASD), and has been associated with poorer functional outcomes, behavioral difficulties, and autism severity across the lifespan. Yet, there is little consensus on best practice approaches to assessing sensory processing dysfunction in adolescents and adults with ASD. Despite growing evidence that sensory symptoms persist into adolescence and adulthood, there is a lack of norms for older age groups, and pediatric assessments may not target appropriate functional outcomes or environments. This review identified approaches used to measure sensory processing in the scientific literature, and to describe and compare these approaches to current best practice guidelines that can be incorporated into evidence-based practice. Method and Analysis: A search of scientific databases and grey literature (professional association and ASD society websites), from January 1987–May 2017, uncovered 4769 articles and 12 clinical guidelines. Study and sample characteristics were extracted, charted, and categorized according to assessment approach. Results: There were 66 articles included after article screening. Five categories of assessment approaches were identified: Self- and Proxy-Report Questionnaires, Psychophysical Assessment, Direct Behavioral Observation, Qualitative Interview Techniques, and Neuroimaging/EEG. Sensory research to date has focused on individuals with high-functioning ASD, most commonly through the use of self-report questionnaires. The Adolescent and Adult Sensory Profile (AASP) is the most widely used assessment measure (*n* = 22), however, a number of other assessment approaches may demonstrate strengths specific to the ASD population. Multi-method approaches to assessment (e.g., combining psychophysical or observation with questionnaires) may have clinical applicability to interdisciplinary clinical teams serving adolescents and adults with ASD. Contribution: A comprehensive knowledge of approaches is critical in the clinical assessment of a population characterized by symptomatic heterogeneity and wide-ranging cognitive profiles. This review should inform future development of international interdisciplinary clinical guidelines on sensory processing assessment in ASD across the lifespan.

## 1. Introduction

Since the incorporation of sensory reactivity into the Diagnostic and Statistical Manual of Mental Disorders (5th ed.; DSM-5), identifying the presence of sensory processing dysfunction has become a key component of diagnosing ASD. Common tools used to diagnose ASD such as the Autism Diagnostic Observation Scale (ADOS) [1] and Autism Diagnostic Inventory–Revised (ADI-R) [2] contain items related to sensory processing, but do not provide specific information about the nature of the sensory processing dysfunction and their impacts on an individual’s daily life [3].

Clinicians who are directly involved in the assessment of the functional, affective, and social impact of sensory processing dysfunction include occupational therapists, psychologists, behavioral analysts, and psychiatrists who may use a range of assessment tools and techniques [4,5,6]. In this age of evidence-based practice (EBP), clinicians face mounting pressure to utilize research evidence when selecting an assessment approach [7]. Evidence-based practice (EBP) is considered to be the “integration of critically appraised research results with the clinical expertise, and the client’s preferences, beliefs and values” [8]. In order to ensure EBP in assessing sensory processing in adolescence and adulthood, clinicians require knowledge of current clinical guidelines, up to date approaches to assessment (e.g., tools and techniques), and current research findings. Yet, to date, no structured review has sought to identify all the potential approaches to assessment and the concepts/terminology used to explain what they are measuring for an adolescent and adult ASD population. As such, three major obstacles to implementing EBP when assessing sensory processing provided the impetus for conducting this scoping review. 

Expanding Transdisciplinary Research Base. Synthesizing an ever-expanding transdisciplinary literature that spans basic neuroscience to clinical intervention can be challenging for clinicians to interpret and use in practice. This is particularly true for the vast literature base pertaining to sensory processing dysfunction where conceptualizations, terminology, and underlying theory may vary depending on discipline [4,6]. Particularly in the areas of neuroscience and physiology, sensory processing dysfunction may be defined narrowly based on sensory channel, peripheral sensation, or neurophysiology, whereas in the clinical sciences sensory processing dysfunction may be more likely to be broadly conceptualized as a combination of observable symptoms that affect behavior or function. 

In a recent conceptual review [6], Schauder and Bennetto (2016) suggest there are two major ways in which sensory processing terminology may become “lost in knowledge translation.” First, certain terms such as “multisensory integration” or “behavior”, may be used in both basic sciences and clinical fields, but have different meanings. Second, a large number of terms are often used interchangeably within the field. These authors suggest that inconsistencies in conceptualizations and terminology may lead to heterogeneity in research findings, further complicating clinical interpretation [6]. Hence, there is a need for a systematic scoping review that incorporates the breadth of sensory terminology and attempts to categorize approaches to assessing sensory processing across both clinical and basic sciences.

Siloed Approaches to Assessment. The National Institute of Clinical Excellence (NICE) in the United Kingdom recommends an interdisciplinary approach to the assessment and management of sensory processing dysfunction in adults with ASD [9,10]. Yet, among clinical disciplines the rationale for, and the approach to, assessing sensory processing dysfunction may differ based on the purpose of the assessment and the desired clinical outcome (e.g., diagnosis, adaptation of the environment, leisure engagement, managing safety), and because different approaches to assessment have been developed within disciplines. Underlying differences in assessment approach are often based on entrenched disciplinary values that may lead to conceptual ambiguities and methodological differences. 

This paper aims to integrate these siloed approaches by scoping a broad body of literature that crosses disciplinary boundaries in order to identify relevant knowledge regarding sensory assessment. A cross-disciplinary analysis may inform clinicians about how and what to assess, and also aid them in understanding the perspectives and methodological contributions of other disciplines that may be using different assessment approaches. This leads to the final clinical challenge–the limitations of existing clinical tools. 

Limitations of Existing Tools. Clinicians typically rely on a combination of direct observation and self- or proxy-report questionnaires when undertaking sensory processing assessments in practice, employed to guide diagnosis and treatment decisions [3]. Yet, as Cascio et al. (2016) [4] suggest, the present methods for measuring sensory processing may not quantify constructs in a valid or reliable way, and underlying theoretical assumptions may require revision. For instance, In relation to pediatric assessment measures, the authors suggest that these parent and self-report questionnaires commonly used in practice and applied in research “often reflect attentional and affective aspects of perception and behavioral response that are less likely to correlate directly with basic sensory processes assessed in the controlled laboratory setting” (p. 923).

More norm-referenced sensory processing measures exist for pediatric populations than for older age groups. Commonly used pediatric measures include the Sensory Processing Measure [11], Sensory Profile (SP) [12], the Sensory Integration and Praxis Test [13], and the Test of Sensory Functions in Infants [14], but these are not normed for adolescent and adult populations. The Adolescent and Adult Sensory Profile (AASP) is a widely used clinical measure for sensory processing in adolescents and adults [15]. Until recently, it had also been the only one with published psychometric properties. 

Despite its widespread use, a number of limitations to the AASP as a clinical tool in ASD have been noted. Elwin et al. (2013) noted that the AASP may miss sensory seeking behaviors in individuals with ASD, where the seeking behaviors tend to be more circumscribed, specific, and repetitive than in the general population. Secondly, individuals with ASD and concurrent intellectual or developmental disabilities (IDD) generally score as “low registration”, although this may not reflect clinical observations. A possible reason for this discrepancy is that the AASP was developed to analyze sensory processing in the general population, and thus the questions may not account for other aspects of cognitive and social functioning that, in addition to sensory processing, might influence behavior. For example, one item on the AASP asks: “I do not get jokes as quickly as others” which may be related to developmentally delayed cognitive or social abilities rather than sensory processing dysfunction. Thirdly, as a self-report questionnaire, the AASP is not designed to be completed by caregivers in situations where an adult with ASD cannot complete the assessment independently. Finally, the AASP does not include questions on interoception, or internal sensory processing, which has been repeatedly demonstrated to be compromised in individuals with ASD and may have significant behavioral implications (e.g., self-injurious behavior, experience of gastrointestinal functions or pain) [16]. Therefore, clinicians need information on other assessment measures or techniques that they could potentially use either alone or in combination with questionnaires like the AASP to address some of the limits of this questionnaire.

While the first two obstacles, challenge clinicians to synthesize the scientific knowledge of potential approaches to sensory processing, and integrate into their clinical assessment techniques, the clinical limitations presented by existing tools are perhaps the most consequential for practice. In order to assess sensory processing in an evidence-based manner, clinicians need to (1) be aware of, and able to navigate the varied terminology and what information different approaches to assessment provide related to sensory processing specific to ASD; (2) be familiar with different approaches to assessment of sensory processing, so that they can effectively work as part of interdisciplinary teams; and (3) have knowledge of a variety of assessment approaches that they can potentially access and use.

Objectives of Scoping Review: This review sought to answer the question: What approaches to assessing sensory processing dysfunction have been applied to date in scientific research with adolescents and adults with ASD and how do they compare to what tools, approaches and rationale for assessment are recommended in clinical guidelines? This scoping review provides a framework from which to compare and critique a wide transdisciplinary literature, and will assist clinicians to consider both the evidence base and potential practicality of using certain approaches. 

The specific objectives of the review were to (1) describe the recommendations regarding sensory processing assessment in clinical guidelines; (2) describe and categorize the scope of sensory processing approaches (i.e., assessment or measure purpose, development, outcomes, and availability) that have been utilized in scientific research pertaining to sensory processing in adolescents and adults with ASD; and (3) Highlight areas of discrepancy between the clinical guidelines and the transdisciplinary scientific literature base.

## 2. Materials and Methods

A review protocol was developed in line with current scoping review reports and recommendations [17,18,19,20,21], originating from Arksey and O’Malley’s detailed methodology [22,23]. A scoping review is “a form of knowledge synthesis that addresses an exploratory question aimed at mapping key concepts, types of evidence, and gaps in research related to a defined area or field by systematically searching, selecting, and synthesizing existing knowledge” [18]. A scoping review methodology was chosen over a systematic review in order to provide a quantitative and qualitative analysis of both empirical and grey literature to an area of research that is complex, broad, fragmented and understudied [20].

The scoping review described in this paper followed five steps [13]: (1) identifying the research question, linking the purpose and research question; (2) identifying relevant studies; (3) using an iterative team approach for study selection and data extraction; (4) charting the data by incorporating descriptive statistics and qualitative thematic analysis; and (5) collating, summarizing and reporting the results, including the implications for practice and research.

This search occurred in two phases. First a comprehensive search of the empirical literature was conducted. Next, based on the findings of this first phase, a systematic search of the grey literature was undertaken. Each phase is discussed in turn.

### 2.1. Phase 1–Empirical Literature Search

Identifying Relevant Studies. The core concepts of interest in this study were “sensory processing”, “ASD” and methods or tools to assess sensory processing. Due to the great variability in sensory terminology, an expansive list of search terms was created. These search terms were based on terminology found in adult sensory processing questionnaires used in practice (i.e., AASP, Sensory Integration Inventory-Revised (SII-R), Analysis of Sensory Behaviour Inventory).

The search strategy was devised to maximize the number of relevant studies included in the review and was created with the assistance of two scientific librarians at the Centre for Addiction and Mental Health (CAMH) in Toronto, ON, Canada and the University of Toronto. The original search terms were utilized to create database-specific Medical Subject Headings (MeSH) headings and key terms. Studies were identified through a systematic literature search based on scoping review recommendations [1,2] of relevant scientific databases, namely MEDLINE, EMBASE, CINAHL, and PsychINFO.

Eligibility Criteria. The inclusion and exclusion criteria are outlined in Table 1. These were developed iteratively by two reviewers. All peer-reviewed research studies available through the CAMH and University of Toronto libraries published between January 1987 (the year before the first known sensory processing assessment was published by Dr. A. Jean Ayres [13]) and May 2017, were included. Non-English language studies were excluded. Further discussion was required as whether to include studies that measured constructs that could be considered to be related to sensory processing. For instance, some studies aimed to measure higher order cognitive constructs such as attention, empathy, or anxiety/hyperarousal, but tested basic sensory processing through neurophysiological or psychophysical methods. In order to be considered a measure of sensory processing, the study had to: discuss at least one aspect of sensory processing dysfunction (i.e., sensory reactivity) as a core concept of interest; describe a subjective or objective method of assessing sensory processing dysfunction in at least one channel of sensory processing; and if a diagnostic tool, have at least ten items related to sensory processing. Once the final inclusion and exclusion criteria had been set, we applied these criteria to the remaining abstracts and all full text screening. Published reviews and commentaries were used as a point of cross-reference to ensure all relevant articles had been found, but since no review or commentary exclusively focused on adults and adolescents, they were not included in the data extraction process.

Article Screening Process. The first 200 titles and abstracts were reviewed independently by the lead (D.D.) and second (E.L.) authors. This process was completed in order to ensure depth of familiarity with terminology, methodologies, and population parameters. The final set of inclusion and exclusion criteria was developed after screening the titles and abstracts. During this preliminary screening process, it became clear that a number of studies, which included adolescents over the age of 16, also included children. In addition, certain databases (CINAHL) would not allow for a specific search of adolescents. Thus, the following revised inclusion criteria were applied: (1) Any study with adolescents/young adults/youth in the title/abstract was included for further review; (2) Studies where adolescents over the age of 16 made up at least part of the sample were included; (3) Any study with child(ren) or boy/girl in the title was automatically excluded from further review. 

After setting eligibility criteria, the reviewers met biweekly to review the remaining articles and resolve discrepancies by consensus. If the study was deemed appropriate for inclusion, or if certain information required for inclusion (e.g., age of participants) was unavailable in the abstract, the full article was reviewed. 

Data extraction. A summary database was created to guide the extraction of data from each empirical research article. The following information was collected from each study: (1) bibliographic information including author, year study was conducted, geographical location, discipline background of first author, study design, purpose; (2) population information including age group, sample size, IQ, ASD diagnosis; (3) scope of information on sensory processing including sensory processing terms used, main findings, and conclusion of the study and (4) approach used to assess or measure sensory processing: including method, development, study design, and clinical availability. 

It should be noted that the intention of this scoping review was not to describe how terminology conceptually differed across assessment methods, but rather to consider how different terminology may impact clinicians’ understanding and use of these assessment measures. For a conceptual review of sensory processing terminology, please refer to Cascio et al., 2016 [4] and Schauder and Bennetto, 2016 [6]. 

### 2.2. Phase 2–Grey Literature Search

Identifying Relevant Studies. An Internet search strategy was developed with a librarian, and conducted in December 2016. Clinical guidelines (Table 2) were identified through an online search of all national ASD organizations in the United States, Canada, United Kingdom, Australia and New Zealand and of the national associations or regulatory bodies in the same countries across major disciplines involved in sensory processing assessment (Occupational Therapy, Psychology including behavioral analysis/cognitive behavioural, and Psychiatry). These countries represent major countries represented in the research literature, as well as the country of publication of this study. Each organization’s website was keyword searched for autism, sensory, adult and adolescent.

Screening Process. Results from the Internet search of associations and regulatory bodies were reviewed by the lead author to identify assessment recommendations and/or guidelines. All information on the webpage was read. Recommendations or guidelines regarding the assessment of sensory processing dysfunction in adults or adolescents with ASD, were cut and pasted into a word document from the webpage. Online documents (PDFs) were downloaded and saved. 

Data Extraction. An excel spreadsheet was created to track: (1) websites and guideline documents that provided recommendations regarding sensory processing assessment, (the organization name, webpage address, and title of document and/or webpage); and (2) specific recommendations regarding assessing sensory processing in adults with ASD. In the event that a clinical guideline was more generally focused on assessing or managing ASD (e.g., across the lifespan; adult ASD diagnoses), only aspects of the document related to assessing sensory processing in adults or adolescents were extracted. 

Data Synthesis of Empirical Studies and Clinical Guidelines. The synthesis generated quantitative and qualitative descriptive summaries of (e.g., frequency and distribution analysis) of the overall dataset, as well the proportion of studies using different assessment approaches, and qualitative analysis of the assessment approach, study purpose, measure development and conceptual or disciplinary basis, and the study findings/conclusions. The data extracted from the empirical studies and clinical guidelines were summarized separately and then compared. Descriptive statistics and proportional tables were used to describe the study characteristics including volume, yearly distribution, and disciplines of the first author and population details (sample size, diagnosis, age ranges etc.). Assessment approach categories were established according to well-known categories of measures, and others that emerged through the review. Data for questionnaires was summarized in a table to facilitate comparison of instruments. 

Qualitative content analysis based on Tricco and colleagues (2016) [17] of grey literature was used (1) to compare current best practices to the assessment approaches utilized in research; and (2) to elucidate methods or approaches to assessment that may not be highlighted in the research literature.

## 3. Results

### 3.1. Empircal Literature Search

The empirical literature search resulted in 4769 citations. After title and abstract screening was completed, 413 full-text studies were reviewed, and data was extracted and charted from 66 studies that fulfilled all eligibility criteria [15,24,25,26,27,28,29,30,31,32,33,34,35,36,37,38,39,40,41,42,43,44,45,46,47,48,49,50,51,52,53,54,55,56,57,58,59,60,61,62,63,64,65,66,67,68,69,70,71,72,73,74,75,76,77,78,79,80,81,82,83,84,85,86,87] (see Supplementary Material for Preferred Reporting Items for Systematic Reviews and Meta-Analyses (PRISMA) flowchart). The grey literature search of 56 websites identified 16 documents or online excerpts presenting clinical guidelines for adults with ASD, and data were extracted from five documents meeting the eligibility criteria. 

### 3.2. Grey Literature Search

Three overarching clinical recommendations were identified through qualitative content analysis: (1) assess sensory processing dysfunction when diagnosing ASD or when sensory processing dysfunction is thought to be related to difficulty with adaptive behavior and/or contributing to maladaptive behavior; (2) if possible, engage in interdisciplinary collaboration when assessing sensory processing, and (3) utilize multiple techniques to assess sensory processing (notably questionnaires, direct observation, and environmental scans of sensory features). 

Clinical guideline documents were from the disciplines of psychiatry, as one of nine areas of possible assessment in the NICE guidelines to a two-page description of how to undertake a sensory environmental scan in the Australian Psychological Association guidelines, and a detailed case study of possible assessment process in the American Occupational Therapy Association (AOTA) guidelines. 

All guidelines made reference to assessing sensory processing dysfunction as a best practice for either the diagnostic or daily management in adults with ASD. Most commonly, they suggested utilizing questionnaires, interviews, and/or direct observation of the person and the sensory environment as assessment approaches, although rarely did they suggest specific tools, or outline how to undertake an observational assessment. The discipline of the clinical guidelines accounted for differences in assessment purpose. For instance, in psychiatry and psychology, the guidelines recommended assessing sensory processing to complete a DSM-5 diagnosis of ASD, and to manage challenging behaviors. The two documents from these fields made several references to conducting functional analyses (structured behavioral observation) in order to understand what sensory triggers might be contributing to challenging behavior. In contrast, the three occupational therapy guidelines were focused more on enhancing daily functioning and participation in occupations such as work, play and education. 

As suggested in the Canadian and American occupational therapy clinical guidelines, by comparing the behavioral responses of the person to the sensory features of common environments, a clinician can better understand how an individual who experiences dysfunction in multiple domains of sensory processing (e.g., hypersensitivity, multisensory integration, and sensory interests) functions in daily life. Observation in real world environments can thus supports clinicians to make clinical decisions about environmental accommodations (e.g., equipment, technology, routines) or modifications (e.g., changes to built environment, recommended environment). 

### 3.3. Empirical Studies 

The study results are first characterized based on date of publication, age range, sample size, population, and study design and rationale. Next each category of assessment approach identified in the review will be described in terms of: specific methods/tools used in the assessment, the assessment purpose, frequency of use within the literature, and key findings. 

**Figure 1:**
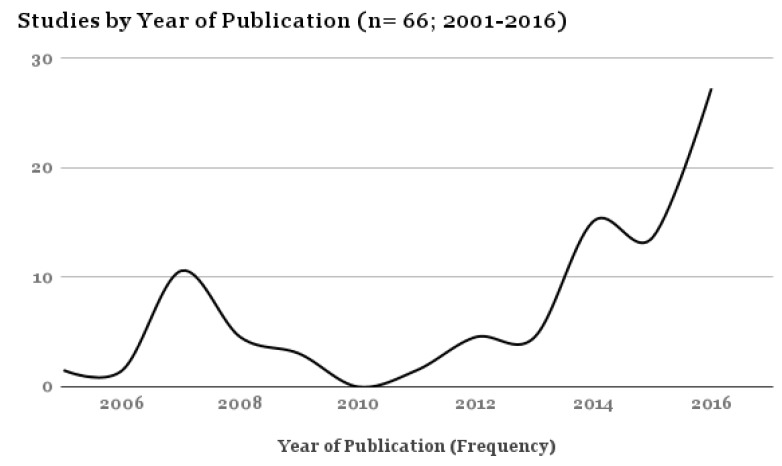
Proportional Table–Studies by Year of Publication.

**Figure 2:**
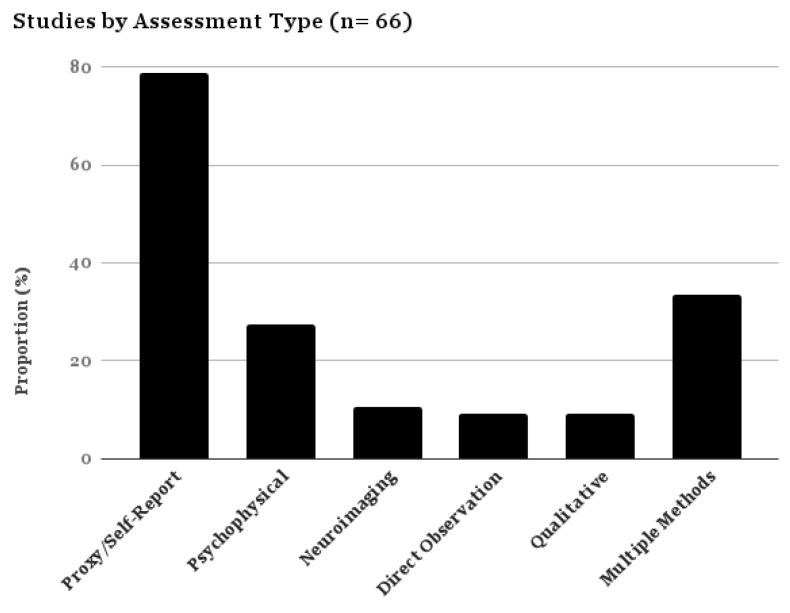
Proportional Table–Studies by Assessment Category.

All articles were published between 2001 and 2017, with 39.46% of the studies (*n* = 27) published in 2016 and the first half of 2017 (Figure 1). Most of the studies were conducted in the United Kingdom (39.39%) and the United States (37.88%). Self- and proxy-report measures were the most commonly used assessment method (78.78% of all studies) (Figure 2).

The increase in published studies over the past year also accounted for a majority of the adult studies. Earlier studies were more likely to include a mixed population (i.e., child, adolescent and adult; or child and adolescent). Fifty-seven percent of the 66 studies focused exclusively on an adult population. 

**Figure 3:**
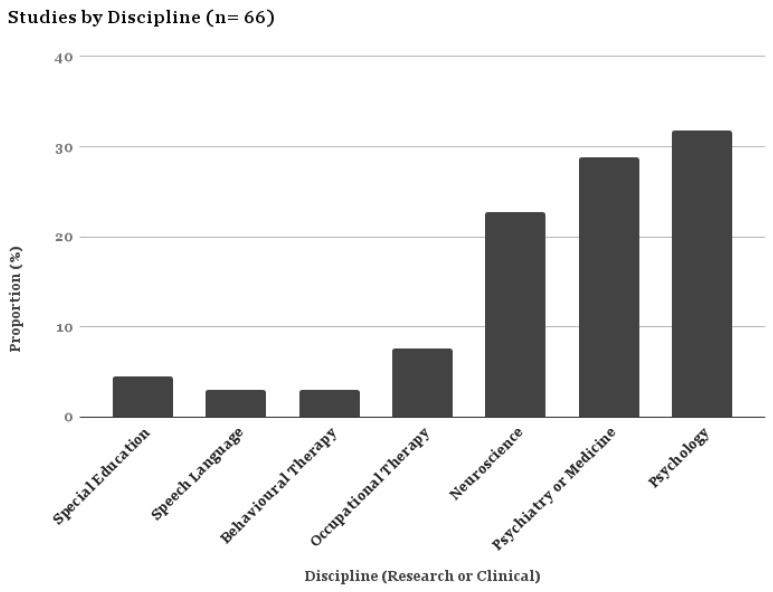
Proportional Table–Studies by Discipline.

Generally the rationale or aims of the studies were either exploratory or measurement-based. The research disciplines of the first author were most often psychology or psychiatry (Figure 3). Exploratory studies aimed to understand (a) the nature of sensory symptoms in ASD; or (b) how sensory symptoms in ASD relate to other constructs (e.g., anxiety or ASD symptom severity) or physiological processes (e.g., proprioception, tactile discrimination, brain activity), while measurement studies aimed to describe the development and psychometric evidence for specific instruments. Four studies evaluated interventions (pharmacological, CBT, sensory integration, and behavioral intervention) and used a sensory processing assessment as an outcome measure.

Cross-sectional (observational) study designs with or without a control group were the most frequent study design (79%). Other study designs included: single case study (9%), qualitative (9%), and quasi-experimental (3%). Study sample sizes ranged from 1 to 800 participants, with 66% of the studies having fewer than 100 participants. Thirty-nine percent of the studies had a range of 5–50 participants.

**Figure 4:**
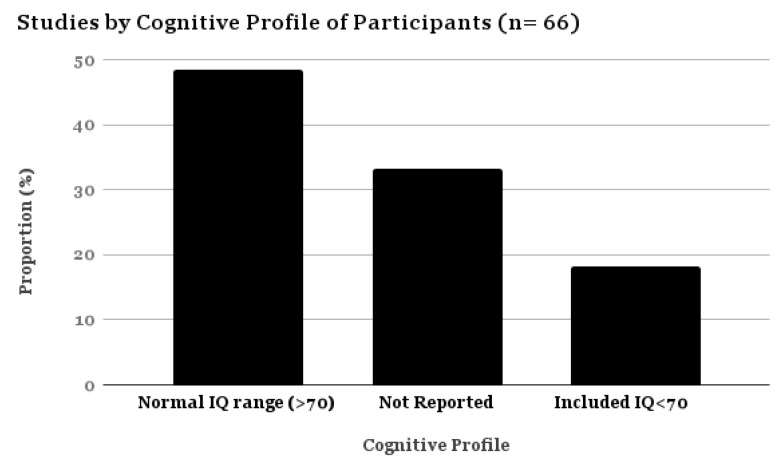
Proportional Table–Studies by Cognitive Profile (*n* = 66).

Only 18.18% of the studies reported including individuals with ASD and a concurrent IDD (Figure 4; also described as “low functioning ASD” or LFASD in the literature). Frequently, studies did not report IQ, but stated they excluded participants with IDD or learning disability. For this review, any study including individuals with an IQ < 70 was categorized as including individuals with IDD. Only seven studies have been conducted exclusively with participants with ASD and IDD, and these studies tended to use single subject designs and aimed to evaluate interventions to reduce challenging behaviors. Also noteworthy is that six out of 31 studies that reported their sample comprised individuals with high functioning ASD (hfASD), had relatively high mean IQs of 110 or above. Gonthier et al., 2016 [45], however, published a relatively large (*n* = 296) cross-sectional study describing sensory processing in adults with ASD and severe IDD.

### 3.4. Summary of Assessment Types

Five separate Assessment Categories were Identified through Data Analysis: Self- and Proxy-Report Measures, Psychophysical Measures, Direct Observation, Qualitative Interviews, and Neuroimaging/EEG.

(1) Self- and Proxy-Report Measures: These are questionnaires requiring respondents to either read the items and select responses, or verbally respond to a list of questions read to them by an interviewer. Questions ask respondents to rate the kinds of difficulties they experience related to sensory processing. Proxy respondents may be caregivers or others, such as a clinician or significant other. Proxy respondents provide responses based on their perceptions of sensory processing behaviors and how frequently these behaviors occur.
(2) Psychophysical Methods: This assessment method quantitatively measures the relationship between a controlled sensory stimulus and an individual’s physiological or self-reported response (e.g., detection, discrimination, and/or comfort thresholds).
(3) Direct Observation: refers to a wide range of techniques (i.e., measure of frequency/intensity of specific behavior pattern, initiation and task completion, motivation and sensory preference assessments, timing, habits/routines, environmental factors) that may be used by clinicians and researchers to capture behaviors indicative of underlying sensory symptoms or preferences in either a controlled laboratory or real-world setting.
(4) Qualitative Interview Methods: These methods involve having individuals describe their sensory experiences in an open-ended or semi-structured interview format, and then the responses are grouped and summarized by a researcher.
(5) Neuroimaging/EEG: Functional neuroimaging measures brain responses through electroencephalogram (EEG) or functional magnetic resonance imaging (fMRI) with the aim to understand the relationship between a brain signal and certain clinical symptoms or observable behaviors.

Each assessment method will be discussed according to purpose, the frequency of use within the literature reviewed, and the findings for each assessment category. Nineteen studies utilized multiple methods to analyze sensory processing. As such, these studies may be represented in more than one category.

#### 3.4.1. Self- and Proxy-Report Measures 

**Figure 5:**
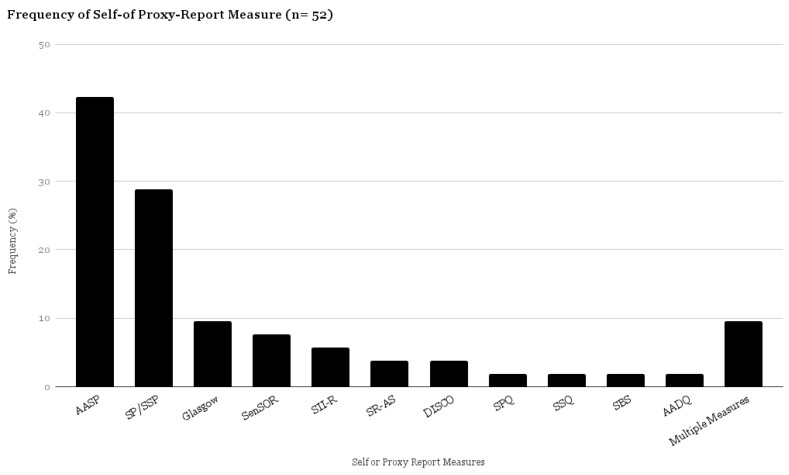
Proportional Table–Questionnaires (*n* = 52).

Eleven questionnaires were identified in this scoping review (Figure 5). A summary table of all identified questionnaires has been provided (Table 3). Questionnaires were primarily used in cross-sectional studies to describe sensory processing patterns in ASD. In regards to the target population, the AASP [88], as well as the Sensory Over-Responsivity Scales (SenSOR) [89], Sensory Processing Questionnaire (SPQ) [81], and SP/Short Sensory Profile (SSP) [12] were developed for use with the general population, while the Sensory Reactivity in Autism Spectrum (SR-AS) [40], Glasgow Sensory Questionnaire (GSQ) [55], the Diagnostic Interview for Social and Communication Disorders (DISCO) [90], Sensory Behaviour Schedule (SBS) [53] and the Sensory Sensitivity Questionnaire (SSQ) [72] were developed specifically to identify sensory processing features of ASD. More than half (54%) of the questionnaires (AASP, GSQ, SR-AS, SPQ, SSQ, AADT) were specifically designed as self-report not proxy-report measures. Of these, the AASP has also been used as a proxy-report measure within the literature with adults with IDD. The AASP, SP/SSP, SII-R, and the DISCO have been used to assess the sensory processing dysfunction in adults with ASD and concurrent IDD, while the SR-AS, GSQ, SPQ, SSQ and SenSOR have only been used in a hfASD population.

All the questionnaires are structured to assess sensory processing across the systems of touch, taste/smell, hearing, and vision, except for the Auditory Attention and Distress Questionnaire (AADQ), which focuses specifically on hearing. The SII-R, GSQ, (SBS) and SR-AS also include proprioception and vestibular as subcategories, and the SenSOR includes proprioception. The AASP/SP, SR-AS, DISCO and the SBS include sensorimotor or kinesthetic processing as a subcategory. Only the AASP and SP have a multisensory (integration) processing category. 

The most common way of conceptualizing sensory processing dysfunction across these sensory systems is using behavioral responses across a continuum from hypo to hypersensitivity. Yet, the terminology conceptualizing this continuum is variable. For instance, while the SPQ and GSQ use “hyper and hyposensitivity”, the SenSOR uses “overresponsivity”, the AASP/SP uses “sensory sensitivity and low registration”, and the SR-AS uses “hypo and hyperreactivity” (and high and low awareness). Not all assessments measured sensory processing on a hypo to hyper continuum; rather, the SII-R, SBS and the DISCO conceptualize sensory behaviors or “symptoms” as either normal or aberrant/abnormal. In addition other questionnaires such as the AASP and the SR-AS attempt to provide a profile or cluster of behavioral features that may contribute to sensory processing dysfunction rather than placing someone on a bidirectional continuum. These questionnaires profile behavior (e.g., sensory seeking or avoiding) and responses to stimuli (e.g., hypo and hypersensitivity). The SPQ was the only identified assessment that focused specifically on basic sensory detection and discrimination abilities. This approach theoretically parallels the psychophysical assessment approach, which will be discussed next. 

As previously mentioned, several questionnaires (GSQ, DISCO, SBS and SR-AS) were developed specifically for individuals with ASD. Thus, the questions focus on sensory processing patterns that are purported to be related to a diagnosis of ASD. Interestingly, the questions included across these ASD-specific questionnaires still demonstrate a range. For instance, the SR-AS queries the categories of strong sensory interest, sensory/motor and interoception, the DISCO includes questions on self-injurious behavior and pain, and the SBS focuses on potential preference for vestibular, proprioceptive and tactile input. 

#### 3.4.2. Psychophysical Methods 

**Figure 6:**
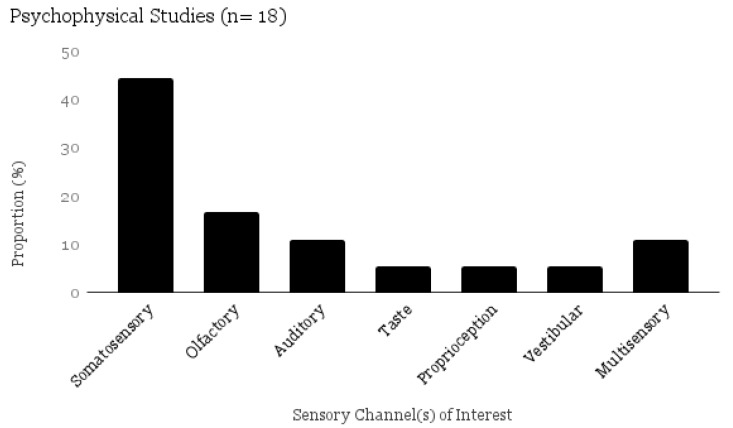
Proportional Table–Psychophysical Methods by Sensory Channel (*n* = 18).

The psychophysical studies included in this scoping review used the following measures to describe sensory processing, detection, discrimination, adaptation, accuracy, reaction time, and/or judgment of intensity and pleasantness of stimuli. Sensation was tested in somatosensory (pressure, vibration, thermal, pain, rough/smooth, proprioception), auditory, olfactory, taste, and vestibular systems (Figure 6).

All studies that implemented a psychophysical method: (a) occurred in a laboratory setting; (b) utilized an objective stimulus; and (c) outlined specific measurement procedures. Frundt and colleagues (2017) [42] have published a study using the greatest variety of psychophysical parameters of somatosensory processing. This study included 13 psychophysical parameters, developed by the German Research Network on Neuropathic Pain of 13 parameters to the hand: cold and warm detection thresholds, thermal sensory limen, paradoxical heat sensations (i.e., participants experienced cold as heat), cold and heat pain thresholds, mechanical detection (MDT), mechanical pain thresholds, mechanical pain sensitivity (sensitivity to pinprick stimuli), pressure pain threshold, vibration detection thresholds, dynamic mechanical allodynia [42].

Most psychophysical studies, however, only assessed sensation in one sensory system (e.g., thermal, pain, taste, smell) using one or two psychophysical methods. Newer studies were more likely to include further parameters. Yet, the stimuli and processes used in psychophysical assessment differed across studies, even when considering the same sensory process (e.g., olfactory, tactile). For instance, three studies tested olfaction [78,85]. In the first, Tavassoli and colleagues (2012) [78] measured threshold and adaptation using Sniffin’ Sticks (n-butanol), Wicker and colleagues (2016) [85] measured suprathreshold detection, intensity judgment, pleasantness judgment, and identification using 24 different common scents, while Addo and colleagues (2017) [24] measured detection, sensitivity, identification, edibility, pleasantness, intensity using 10 scents. 

#### 3.4.3. Direct Behavioral Observation

Observation can be collected by naturalistically observing daily routines/participation in the individuals’ natural environment, or in a controlled setting, for instance, during the course of a functional behavioral assessment (FBA). How the term “sensory” is applied in an FBA is specific to the observable behavior of concern, rather than as a representation of the underlying neurophysiological deficits within the sensory system. For example, the focus would be on behaviors such as repetitive movements, spinning, rather than detection or intensity of sensations within vestibular or tactile systems. The studies that utilized observation within this scoping review generally used an FBA as an outcome measure within a single case study design. Within behaviorism, an FBA is utilized to determine what may trigger and maintain a behavior, commonly used in the assessment of individuals who have severe cognitive or communication impairments [54]. Behavior is seen to be maintained by social (attention, tangibles, escape/avoiding) or non-social reasons (automatic/sensory). When a behavior is seen to be reinforced by automatic, or ‘sensory’ reasons, it is considered non-social, and often involves restricted, repetitive and/or self-stimulatory behaviors [54]. 

Two of the studies implemented sensory preference assessments [54,73]. Preference assessments are conducted within FBA to identify an individual’s favorite inputs to be used as reinforcers or replacements during interventions designed to decrease self-injurious behavior. In each of the studies, sensory inputs were provided in either a structured array or in competing trials. Those items that were most highly preferred (more than 80% engagement with item, less than 20% engagement in self-injury) were then utilized as an aspect of intervention. 

#### 3.4.4. Qualitative Interview Techniques 

Qualitative interviews were used to elicit first person accounts of sensory experiences/preferences from individuals with ASD, and were used with or without other self-report measures. The studies in this review utilized qualitative methods to expand conceptualizations of sensory processing (dysfunction) in order to develop new measures or to explore how sensory processing might impact function or participation. The approach to qualitative inquiry included open-ended or semi-structured interviews or focus groups either alone [39,67,75] or in combination with a questionnaire [25,32,56]. These interviews asked about compensation strategies, sensory interests, daily routines and experiences of sensory processing dysfunction in specific environments such as work or school, and internal sensations/feelings. Results were analyzed through content or thematic analysis. Qualitative analysis was used in the development of two ASD-specific questionnaires—the SR-AS [39,91] and the GSQ [75]. 

#### 3.4.5. Neuroimaging/EEG

Neuroimaging has been used to advance knowledge of the underlying neurophysiology associated with sensory processing dysfunction in ASD, and to establish theoretical propositions for sensory behaviors. Techniques such as functional magnetic resonance imaging (fMRI) during resting state or experimental paradigms, and electroencephalogram (EEG), which measure the electrical activity of the brain, have been used to explore potentially aberrant neuronal processing in individuals with ASD. 

Seven neuroimaging studies were included in this scoping review. The purpose of these studies was to test the neurological underpinnings of specific aspects of sensory processing dysfunction in ASD, such as multisensory integration (e.g., combined auditory and visual), reaction time, perceptual load, or habituation. Three studies by Green and colleagues combined fMRI and clinical questionnaires [46,47,48]. Two of these measured sensory over-responsivity (SOR) with the SenSOR, or a combination of the SenSOR and SSP and correlated these findings with Blood Oxygen Level Dependent (BOLD) signals [46,47].

## 4. Discussion 

The primary objective of this scoping review was to identify and characterize approaches to assessing sensory processing that have been used, in research, with adults or adolescents with ASD. This review also aimed to describe recommendations for assessment made in clinical guidelines, and to compare to study and population characteristics and scope of information available on each assessment approach. 

The 66 empirical studies included in this review, were categorized according to assessment approach: Self- and Proxy-Report Measures, Direct Behavioral Observation, Qualitative Interviews, Neuroimaging, and Psychophysical Assessment. As demonstrated in the results, sensory research to date has focused on individuals with high-functioning ASD, most commonly through the use of self-report questionnaires. The Adolescent and Adult Sensory Profile (AASP) is the most widely used assessment measure, however, a number of other assessment tools and techniques may demonstrate clinical strengths for use with an ASD population. 

This scoping review demonstrated that self or proxy-report measures were the predominant assessment category used in research measuring sensory processing in adults/adolescents with ASD, with 78.7% of the studies using this approach. Of these, the Sensory Profile set of assessments comprised a significant margin, being used in 71.1% of studies that used a questionnaire method. The studies published in late 2016 and early 2017, however, have applied other questionnaires [40,41,74,84]. The major clinical strength of self- and proxy-report measures is the ease of implementation and breadth of information that may be collected related to different aspects of sensory processing. Information can be collected in a relatively short period of time, and may not even require the person to be physically present (e.g., online/telephone). 

As demonstrated in Table 2, although all questionnaires focus on the core construct of sensory processing, they did not all conceptualize this the same way. Therefore, in order to thoroughly assess an adult with ASD, it may be necessary and preferable to triangulate the results from multiple questionnaires, or to use different questionnaires depending on the purpose of the assessment [3,4,6,16]. For instance, some self-report measures (e.g., the SPQ) focus on assessing basic sensory perception. (e.g., discrimination, adaptation, thresholds), whereas other instruments (e.g., the SR-AS, SBS, DISCO) assess sensory symptoms/behaviors common in adults with ASD, and are more closely aligned with a diagnostic purpose. The SPQ, particularly when used in conjunction with psychophysical assessments, may provide a useful clinical picture of the discrepancies in basic perception and self-reported experience of (peripheral) sensory input, which has been shown to be abnormal in ASD [92]. Assessing these specific discrepancies has direct functional implications related to safety (temperature/pain; falls; accessing healthcare; dressing; bathing) as well as possible interventions (e.g., body awareness training, desensitization therapy). 

Questionnaires also have some major limitations for clinical use. First, it can be challenging to choose what questionnaire to use. For clinicians who do not have a strong theoretical knowledge of sensory processing, choosing the appropriate questionnaire may be confusing, particularly if the assessment does not have a published instruction manual. Despite identifying 11 questionnaires, only three of these are both norm-referenced and clinically-available which means the clinicians decisions of what tool to use are already significantly narrowed. Another challenge in deciding on a questionnaire is the type of information it does or does not deliver. Internal sensation processing, which may have significant implications for biological processes such as pain and gastrointestinal functioning, is not reflected in the available sensory questionnaires. The questionnaires identified in this scoping review have by and large been developed and validated with high functioning ASD individuals who can self-report. Only the Sensory Profile group of assessments and the DISCO have published results for individuals with ASD and a concurrent IDD. In the case of the AASP/SP, this required the tool to be used in a non-standardized way, potentially limiting the validity of the results. 

To our knowledge, this is the first review to systematically identify instruments to assess sensory processing in adolescents and adults demonstrating the range of options other than the AASP. It should be noted that this list does not include questionnaires that may be available for clinical use, but have not been studied in the population of interest. For instance, given that some pediatric assessment measures, such as the SP/SSP and SenSOR have been applied to an adult population [61,62,63,64,81], it was somewhat surprising that certain questionnaires such as the SIPT, and the Analysis of Sensory Behaviour Inventory had not been used in research with adults. Given the plethora of research that has been published in the past year alone, it is an ideal time to conduct a systematic review of the identified questionnaires, as there is no evidence synthesizing and comparing their psychometric evidence. 

The widespread use of the Sensory Profile group of assessments is likely attributable to the fact that it puts forth a behavioral and neurological model for typical and abnormal sensory processing, and has long been one of the few norm-referenced measures of its kind available to researchers. Despite its apparent functionality in a research context, the clinical relevance of this group of assessments is limited when working with adults with ASD and concurrent IDD [39,45]. Thus, more research is required before a gold standard sensory processing questionnaire for adults with ASD can be identified. A number of questionnaires, in particular the SR-AS, have been developed based on the experiences of individuals with ASD. Clinicians could consider adding these newer tools to their clinical practice, recognizing that that they are still in early stages of development. Future research should focus on questionnaires developed specifically for individuals with ASD, through consultation with individuals with ASD.

While questionnaires provide structure and may provide normative scores, qualitative techniques such as semi-structured questions used in clinical interviews are a more flexible method for gathering clinically relevant information on a range of topics, and the clinical interview commonly featured into clinical guidelines. The open-ended approach may allow for more individualization, which captures client-specific information on how sensory processing dysfunction impacts his/her daily life. Individuals may also feel more comfortable sharing information in an interview context. Based on the communication abilities of the client, this method may also be more inclusive, and allow for flexibility in how questions are approached. For instance, the use of visual aids, “follow along”, and questions about impact on meaningful activities and environments can be more easily incorporated and may support the conceptualization of sensory processing for individuals who are non-verbal. Use of these techniques may be considered in future research. The clinical interview, however, is limited by the skills of the clinician, requires a strong theoretical understanding of sensory processing in adults with ASD, and can still be challenging and time consuming to implement with adults with ASD who have difficulties communicating. 

All clinical guidelines suggest that observation should be a key component of assessing sensory processing, particularly for adults with ASD and concurrent IDD who may not be able to self-report. Observation, however, can take different forms, and in this review, observation was constricted to a specific behavior (e.g., skin picking) or physiological response to stimuli presentation of stimuli (tactile, visual, auditory, olfactory) under lab-based conditions. Limited research exists describing how to undertake a clinical observation related to sensory processing in real world environments. In order to comprehensively assess sensory processing patterns in adolescents and adults, a combination of naturalistic and structured observations may be required. Naturalistic observation should include leisure, activities of daily living /social interactions in familiar and unfamiliar home and community environments, while structured observation should occur in a safe, familiar and/or contained environment where a range of sensory stimuli were presented, as well as during performance of daily tasks or activities. The Sensory Processing Scale Assessment [93] includes a structured observation component, but has not been tested in an adult or adolescent population.

The FBA approach dominated the observation category in the research literature. Yet, the terminology (e.g., automatic/sensory, repetitive and restricted behaviors) and principles of behaviorism, that underlie this approach, do not account for the neurophysiological aspects of sensory processing dysfunction in ASD. Thus, a major gap in the literature exists: while behavioral approaches may either minimize or exclude a consideration of the neural and physiological factors that might explain behavior related to sensory dysfunction, neural and psychophysical tests may neglect the person/environment relationships. 

To contend with this gap in knowledge related to the interrelationships between neurophysiological, behavioral and environmental influences, more guidance would be useful for clinicians regarding how to perform clinical observation as a standard aspect of a sensory processing assessment. Future studies that combine structured observation across environments with other assessment methods (e.g., questionnaires, interviews, psychophysical methods) would be useful for clinicians. Currently little guidance is available in the research on how multiple domains of sensory processing dysfunction are represented in an individual and how this influences interaction with the environment. Structured guidelines on what to observe and, in particular, how to engage in a sensory preference assessment (e.g., presentation of a structured array of sensory input) may ensure a minimum assessment standard and would potentially limit bias in process and interpretations. Such assessment standards may allow for clinical recommendations that are individualized, safe, and effective. 

Psychophysical methods were not suggested within any of the reviewed clinical guidelines as a component of sensory processing assessment for adults with ASD. Despite this, they are being applied with increasing frequency in the research literature, used in about a quarter of all studies. Yet, there is still no gold standard on how psychophysical features in individuals with ASD should be assessed in research or clinically [42]. Psychophysical methods may provide clinically-relevant information about baseline detection of sensory information that may improve clinical decision-making and compliment the information gathered through self-report questionnaires. For instance, the Sensory Challenge Protocol [94] may be useful for assessing hypo or hyperresponsivity to sensory stimuli, though no studies have been published on its applicability to an adult population. Several limitations to psychophysical methods may account for its infrequent use in comparison to questionnaires. These limitations include: the lab-based nature of the tasks, access to costly technology, specialized knowledge of testing procedures, and a narrow scope (e.g., specific sensory channel). In addition, it may be challenging to implement tasks that require a standardized administration procedure, with individuals with ASD who have difficulties communicating. 

Neuroimaging also currently has major limitations to clinical use due to cost, availability, training and insensitivity of resting-state results. Currently, neuroimaging is used in research to advance knowledge rather than to diagnose or guide the clinical management of sensory dysfunction. In the future, however, brain imaging may be utilized in combination with other assessment methods, to hone in on specific brain networks or structures to target to influence functioning. The clinical applicability of neuroimaging is likely to alter in the near future, as interventions that target neural or physiological processes such as repetitive transcranial magnetic stimulation, deep brain stimulation, medication/hormone therapy, desensitization therapy, and biofeedback applications become more commonly integrated with clinical practice [16]. 

### 4.1. Use of Multiple Methods

Currently, there is no single clinical assessment tool that can assess all aspects of sensory processing dysfunction, and each approach to assessment has advantages and limitations for use in clinical practice [3]. Given that different categories also represent different disciplinary expertise (e.g., neuroimaging is a specialized skill set requiring technical resources and training), interdisciplinary collaboration is required and recommended by available clinical guidelines. This review may facilitate collaboration of multiple disciplines by naming the various methods and providing assessment categories that cross disciplinary boundaries. Yet, less than one third (31%) of studies in this scoping review combined assessment approaches across two separate categories of measurement. Since the publication of the DSM-5 there has been a notable increase in studies looking at sensory dysfunction. The updated diagnostic criteria and other factors such as an evolving knowledge base and greater access to technology may have contributed to comparing the findings of self-report questionnaires with other approaches to assessment (e.g., direct observation, or neuroimaging). 

Within research, triangulation using multiple approaches to assessment may improve understandings of the cognitive and neurological bases of sensory perception in ASD and how individuals function in their daily life. There is great potential for these types of studies to inform theory, and enhance interdisciplinary conceptualizations and approaches to assessment and intervention. 

### 4.2. Implications and Future Directions

A number of recommendations for clinical practice and future research are proposed, based on the findings of this scoping review. 

Conduct a systematic review on psychometric properties. The results of this scoping review demonstrate that due to the development of several new questionnaires over the past decade, a systematic review to compare the psychometric properties and results of these questionnaires is warranted. To date, no systematic review has been published on the measurement properties of sensory processing questionnaires used with adult or adolescent populations.

Develop international interdisciplinary clinical guidelines. We recommend that an international interdisciplinary expert conference on sensory processing in ASD (and IDD) take place to develop clinical guidelines for assessment across the lifespan. This forum may also act as a knowledge translation bridge in communicating about sensory processing research across disciplines. As envisioned by Cascio et al., 2016 [4], “outcomes of successful cross-disciplinary collaboration will allow for better translation of empirical finding to clinical practice and improved assessment and interventions with persons with ASD” (p. 924). 

Embed sensory processing knowledge into clinical education. The over-reliance on questionnaires to assess sensory processing in ASD may be improved by embedding basic sensory processing and ASD content into clinical curricula. In addition, it may be useful to develop and offer an advanced certificate/training program specific to sensory processing in ASD for interdisciplinary teams or clinicians who practice in this area. Both NICE [9] and the CAOT (2015) call for increased focus on autism and sensory processing in health science education. Experience in basic sensory-perceptual testing (more commonly used in pediatric or neurology settings) may also provide clinicians working with adults with ASD with the skills to use psychophysical methods. Hyporesponsiveness in particular may be more directly and accurately captured by psychophysical methods given that these symptoms are defined as the absence of typical responding [6].

Conduct interdisciplinary, multidimensional assessments that triangulate results. No one assessment can analyze all aspects of sensory processing dysfunction. Where sensory processing is considered a major feature of ASD with serious behavioral and functional implications, multidimensional assessment combining direct (systematic) observation, self or proxy-report measures, interviews, psychophysical, and potentially neuroimaging methods, ensures a comprehensive evaluation. The use of interdisciplinary teams, and the incorporation of clinician-scientist positions increases the feasibility of combining multiple methods. For individuals with impaired communication abilities, direct observation should include an interactional component where the individual is provided with sensory materials or is observed engaging in different sensory environments, termed a sensory preference assessment. In order to be a clinically relevant, systematic assessment of all sensory channels should be included. Interdisciplinary collaboration among occupational therapists, psychiatrists, psychologists, behavior therapists, and other professions will allow for more complex, multidimensional assessments. 

### 4.3. Future Methodological Considerations

The studies to date represent a wealth of information. Yet several research gaps exist. For instance, a well-designed multidimensional study to characterize sensory processing in adults with ASD has yet to be published. In addition, potentially due to the biomedical framing of the papers, the role of the environment in shaping how an individual with ASD may experience sensory processing dysfunction has played a minimal role in the current research. Finally, few studies include individuals with IDD in their samples limiting the clinical applicability to a LFASD population. A major impetus for undertaking this scoping review was clinical obstacles to utilizing existing tools with clients with ASD and concurrent IDD. Despite completing an exhaustive review of the literature, only 17% of the studies included individuals with IDD in their sample. As described by DuBois et al., (2016) [16], advances in communication technology have demonstrated that many individuals with ASD who have communication difficulties may be able to provide feedback about physiological processes, if adapted to meet their communication and sensory needs. Incorporating the use of communication aids and adapted communication strategies such as pictures and visual scales should be included in future research.

### 4.4. Study Limitations

There were several limitations to this scoping review. Only English language studies were included which means other assessment approaches/instruments published in other languages may have been missed. In addition, there are other databases cataloguing research articles related to ASD that were not included in the search strategy and therefore it is possible that other articles could have been included in this study. Finally, a wider grey literature search may have led to inclusion of more clinical practice guidelines. The grey literature phase also did not undergo a consensus review by a second reviewer.

## 5. Conclusions

This review identified a number of limitations relating to the use of self- and proxy-report measures as a single approach in this population. Future studies should consider using a variety of assessment approaches that combine measures and techniques. There is also a need to establish assessments designed for adolescents and adults with ASD and to account for the prevalence of concurrent IDD in this population.

## Figures and Tables

**Table 1:** Empirical Studies–Eligibility Criteria.

*Inclusion Criteria*	*Exclusion Criteria*
-Original research published in peer-reviewed journals	-Non-peer-reviewed
-Available electronically through CAMH or University of Toronto Libraries	-Did not focus directly on sensory processing (e.g., ASD diagnostic tools with limited sensory processing questions)
-Diagnosis of ASD with or without an IDD.	-Non-English language
-Use of a clearly defined sensory processing measure or technique	-Theses and conference abstracts
	-Publication date before 1988
-Age 16+ included in study sample	-Reviews and Meta-analyses (used for cross-reference)

**Table 2:** Included Clinical Practice Guidelines.

Organization	Country	Title	Year
National Institute of Health Care and Excellence	United Kingdom	Recognition, referral, diagnosis, and management of adults with autism	2012
Australian Psychological Association	Australia	Clinical Assessment Resource: An overview of best practice tools and approaches to conducting biopsychosocial and developmental assessment of child, young people, and adults with a disability who display behaviors of concern	2011
Canadian Association of Occupational Therapists (CAOT)	Canada	Position Statement on Autism Spectrum Disorders and Occupational Therapy	2015
American Occupational Therapy Association (AOTA)	United States	Scope of Occupational Therapy Services for Individuals with ASD across the Life Course	2015
College of Occupational Therapy	United Kingdom	Occupational Therapy and Learning Disabilities	2012

**Table 3:** Sensory Processing Questionnaires Applied to Adults/Adolescent ASD.

Self- or Proxy-Report Measures	Creators	Subscales/Items	Terminology	Age Group	Scoring	Clinically Available (Y/N)	Published Psycho-metric Prop-erties (Y/N)	Development	Discipline	Self- or Proxy-Report	ASD Specific (Y/N)
AASP	Dunn, 2001	Low Registration, Sensory Sensitivity, Sensory Avoidance, Sensory Seeking; taste/smell, movement, visual, touch, multisensory, activity level, and auditory	Four Quadrant Model of Sensory Processing; Neurological and Behavioral Thresholds	11+	60 items; 4 point scale	Y	Y	Expert panel, factor analysis, construct validity–physiological measure	Occupation-al Therapy (OT)	Self-Report	N
SP/SSP	Dunn, 1997	Sensory processing, modulation, and behavioral and emotional responses; taste/smell, movement, visual, touch, multisensory, activity level, and auditory	Four Quadrant Model of Sensory Processing; Neurological and Behavioral Thresholds	3–10	125 items; 38 items (short); 4 point scale	Y	Y	Expert panel, factor analysis, construct validity–physiological measure	OT	Proxy-Report	N
SenSOR	Schoen et al., 2008	Sensory over-responsivity (sensory sensitivity or sensory avoiding); touch, vision, hearing, smell, taste, and proprioception	Sensory Modulation	All ages	Unclear	N	Y	Factor analysis (discriminant validity, internal reliability/consistency); ongoing development; expert and lit review; correlated to examiner-scored behavior analysis and AASP	OT	Self/Care giver- Report	N
SII-R	Reisman & Hanschu, 1992	tactile, vestibular and proprioceptive processing; “general reactions” section	Sensory Integration	Adult	Checklist	Y	N	Based on sensory integration theory	OT	Proxy-Report	N (IDD Specific)
GSQ	Horder et al., 2014	visual, auditory, gustatory, olfactory, tactile, vestibular, and proprioceptive	Hypo and Hypersensitivities	Adults with ASD	42 items; 4 point scale; total score	N	Y	Factor analysis across cultures; reports in the literature of sensory signs and symptoms commonly associated with ASD and b signs and symptoms reported by parents of children with ASD; correlated to AASP	OT	Self-Report	Y
SR-AS	Elwin et al., 2016	hyper- and hypo-reactivity, strong sensory interest, and sensory/motor; visual, auditory, gustatory, olfactory, tactile, vestibular, proprioceptive and interoceptive	High and low awareness	Adults with ASD	38 items; 4 point scale	N	Y	Qualitative interviews; factor/cluster analysis	Psychiatry	Self-Report	Y
DISCO	Wing, Leekam, Libby, Gould, & Larcombe, 2002	touch, taste, smell, kinesthetic, auditory, and visual. Items relating to atypical taste/oral, movement, touch responsiveness, and self-injurious behavior	“proximal” (e.g., smell, taste, touch, kinesthetic and mixed)	Mixed Age Group–Autism	25 items (sensory); 300 total for diagnostic tool	Y	Y	Chosen based on clinical observation; developed as part of diagnostic tool; factor analysis	Psychology	Proxy-Report/Inter-view	Y
SPQ	Tavassoli et al., 2014	touch, hearing, vision, smell, and taste	basic detection and discrimination to sensory input measured across hyper-and hyposensitivity	Adults	35 item; 4 point scale	N	Y	Factor analysis; correlated to SenSOR; developed to study basic detection and discrimination–based on ‘main sensory modalities’ (Goldstein, 2002)	Psychiatry	Self-Report	N
SBS	Harrison & Hare, 2004	visual, auditory, gustatory, olfactory, tactile, vestibular, movement, proprioception, temperature and sensory processes	sensory symptoms (ongoing, past, none)	Adults with ASD	17 items; ongoing, past, none	Y	N	Literature review, discussion with experts, and based on O'Neill (1995)	Psychology	Proxy-Report	Y
SSQ	Minshew & Hobson, 2008	sound, light, tactile and temperature sensitivities, pain tolerance, awareness of smell or taste, and sensitivity to environmental events or conditions	sensory sensitivity	Mixed Age Group	13 item; Yes/No	N	N	Based on items from the highly sensitive person self-report checklist (Aron and Aron 1997), common reactions to sensory stimuli reported by individuals with ASD, classic behavioral descriptions, and clinical reports	Psychiatry	Self-/Proxy-Report	Y
AADQ	Dunlop et al., 2016	Auditory	auditory hypersensitivity; difficulty hearing in noisy environments	Adults	33 items; 7 point Likert scale	Y	N	Based on validated inventories for specific adult clinical populations that experience abnormal auditory processing	Speech Language	Self-Report	N

## Supplementary Materials

The supplementary file is available online at www.mdpi.com/2076-3425/7/8/108/s1.
