# Supplementary figures and images for: Assessing Sensory Processing Dysfunction in Adults and Adolescents with Autism Spectrum Disorder: A Scoping Review
Source: Brain Sci. 2017 Aug 19;7(8):108. doi: 10.3390/brainsci7080108 (PMC5575628; doi:10.3390/brainsci7080108)

## SUPPLEMENTARY FILE

## PRISMA Flowchart

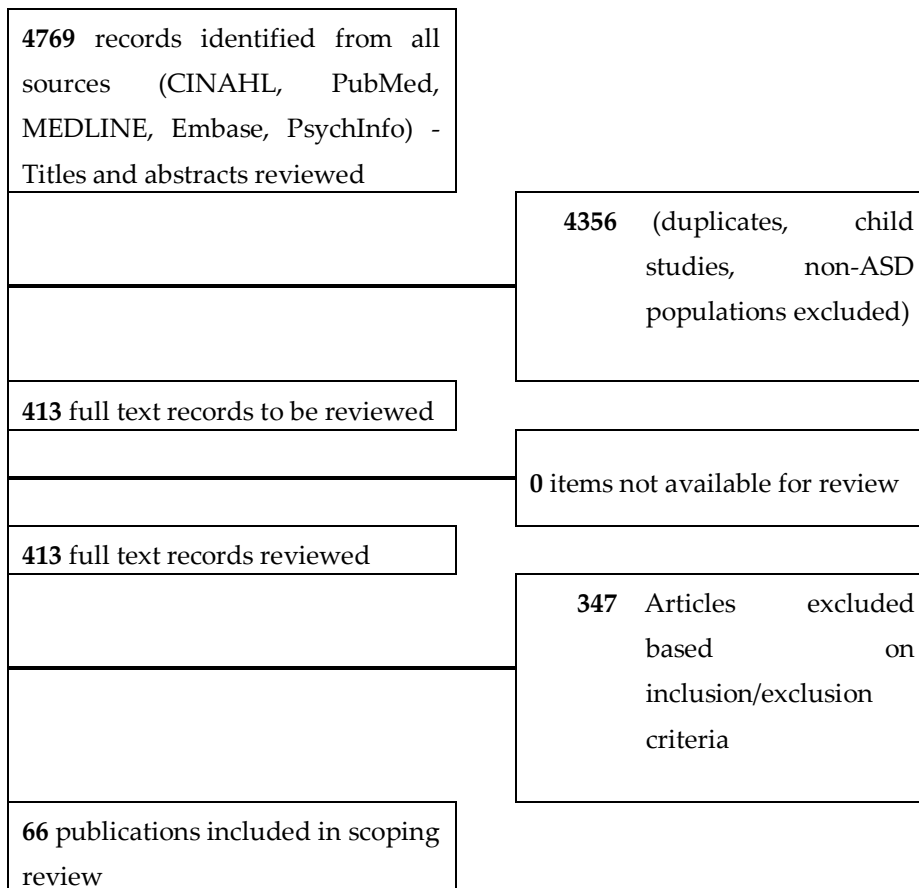

Supplement: Supplementary file 1 [file brainsci-07-00108-s001.pdf]
